# Unexplained Falls Are Frequent in Patients with Fall-Related Injury Admitted to Orthopaedic Wards: The UFO Study (Unexplained Falls in Older Patients)

**DOI:** 10.1155/2013/928603

**Published:** 2013-02-25

**Authors:** Mussi Chiara, Galizia Gianluigi, Abete Pasquale, Morrione Alessandro, Maraviglia Alice, Noro Gabriele, Cavagnaro Paolo, Ghirelli Loredana, Tava Giovanni, Rengo Franco, Masotti Giulio, Salvioli Gianfranco, Marchionni Niccolò, Ungar Andrea

**Affiliations:** ^1^Geriatric and Gerontology Institute, University of Modena and Reggio Emilia, Modena 41121, Italy; ^2^Geriatric Department, Azienda Policlinico Federico II, Naples 80131, Italy; ^3^Unit of Gerontology and Geriatric Medicine, Department of Critical Care Medicine and Surgery, University of Florence and Azienda Ospedaliero Universitaria Careggi, Florence 50134, Italy; ^4^Geriatric Unit, Santa Chiara Hospital, Trento 38122, Italy; ^5^Department of Geriatrics, Azienda Sanitaria Locale 4, Chiavari 16043, Italy; ^6^Division of Geriatrics, Ospedale S Maria Nuova, Reggio Emilia 42123, Italy

## Abstract

To evaluate the incidence of unexplained falls in elderly patients affected by fall-related fractures admitted to orthopaedic wards, we recruited 246 consecutive patients older than 65 (mean age 82 ± 7 years, range 65–101). Falls were defined “accidental” (fall explained by a definite accidental cause), “medical” (fall caused directly by a specific medical disease), “dementia-related” (fall in patients affected by moderate-severe dementia), and “unexplained” (nonaccidental falls, not related to a clear medical or drug-induced cause or with no apparent cause). According to the anamnestic features of the event, older patients had a lower tendency to remember the fall. Patients with accidental fall remember more often the event. Unexplained falls were frequent in both groups of age. Accidental falls were more frequent in younger patients, while dementia-related falls were more common in the older ones. Patients with unexplained falls showed a higher number of depressive symptoms. In a multivariate analysis a higher GDS and syncopal spells were independent predictors of unexplained falls. In conclusion, more than one third of all falls in patients hospitalized in orthopaedic wards were unexplained, particularly in patients with depressive symptoms and syncopal spells. The identification of fall causes must be evaluated in older patients with a fall-related injury.

## 1. Introduction

Falls in older people are a major public health concern in terms of morbidity, mortality, and health and social services costs [1].

Falls are the leading cause of injury-related visits to emergency department in the United States. Trauma is the fifth leading cause of death in people starting from 65 years, and falls are responsible for 70% of accidental death in people starting from 75 years.

More than a third of older adults falls each year [2]. About one-third of community-dwelling elderly people and up to 60% of nursing home residents fall each year; one half of these “fallers” have multiple episodes [3]. Nearly all hip fractures occur as a fall result [4]. Fall-related injuries among older adults, especially among older women, are associated with substantial economic costs, mostly because of hip fractures and their subsequent disability [5].

Data regarding fall types in patients admitted to orthopaedic wards because of fall-related injury are lacking: the UFO study (Unexplained Falls in Older Patients) was made to assess the incidence and the clinical characteristics of unexplained falls in this specific group of elderly subjects affected by fall-related fractures. 

## 2. Methods

### 2.1. Definition of Fall

We defined four different types of falls: “accidental” (fall explained by a definite accidental cause), “medical” (fall caused directly by a specific medical disease, e.g., hypoglycemia, drugs, drop and attack, transient ischemic attack, myocardial infarction, arrhythmic drugs, orthostatic hypotension), “dementia-related” (fall in a patient with previous diagnosis of moderate-severe dementia), and “unexplained” (nonaccidental falls, not related to a clear medical or drug-induced cause, where no apparent cause has been found) [6].

### 2.2. Protocol

All enrolled patients were starting from 65 years and consecutively admitted to orthopaedic wards because of fall-related injury, without any exclusion criteria. 

All patients (or relatives if the patient had diagnosis of dementia) gave informed written consent. 

Centers involved in the study (the appendix) designated and instructed a trained investigator who used to manage falls and syncope to run the study.

All subjects were asked to complete their clinical history, with a specific questionnaire about fall characteristics, pharmacologic anamnesis considering all drugs taken in the last month, clinical and neurological examination, routine blood chemistry tests, and 12-lead ECG. 

Moreover, we performed a multidimensional geriatric evaluation including Mini Mental State Examination-(MMSE) [7] to assess cognitive performance, Geriatric Depression Scale (GDS) [8], to screen the presence of affective disorders, basal (BADL) [9] and instrumental (IADL) activities of daily living [10], to evaluate disability, and Cumulative Illness Rating Scale to define comorbidity (CIRS) [11].

### 2.3. Statistical Analysis

Data analysis was performed using SPSS, 14th version (SPSS, Chicago, IL, USA). The *χ* ^2^ test was used to compare proportions in univariate analysis of dichotomic variables and to calculate odds ratio and the 95% confidence intervals. Student's *t*-test for independent samples was used to compare continuous variables. Variables significantly associated with the outcome of interest in univariate analyses were entered into a multivariate logistic regression model (backward stepwise) to assess their independent association with the outcome. A *P* value <0.05 was considered statistically significant.

## 3. Results

246 patients (mean age 82.3 ± 7.2 years, 82% females) were submitted to the basal evaluation. We divided patients into two groups, according to age: 65–79 years (*N* = 76), ≥80 (*N* = 159). Most patients (*N* = 161) were admitted because of a fall-related hip fracture.

Clinical characteristics of the studied sample are shown in Table 1. 

Patients older than 80 years were more likely to be self-dependent and obtained lower MMSE scores; they were more likely to show depressive symptoms, and they had lower values of BMI. No differences were found in the two groups in terms of biochemical values, except for hemoglobin that was significantly lower in older subjects. 17 patients (8.1%) had syncope as a cause of fall. According to the anamnestic features of the event, older patients had a lower tendency to remember the fall (Table 2).

Data regarding drugs taken in the last 30 days are shown in Table 3: 184 of 246 enrolled patients were taking at least one drug (74.7%). Older patients were more likely to take diuretics, and no other difference was found between the two groups.

## 4. Fall Types

The different fall types are described in Table 4.

Younger patients had a higher number of falls documented as accidental (48.1% versus 36.5%, *P* = 0.02), while older patients were more frequently affected by dementia, as expected. No other differences were found for the other fall types (Table 4). 

Clinical characteristics of patients with different fall types are shown in Table 5. Patients with dementia-related falls were significantly older than patients with accidental falls (85.9 ± 1.2 versus 80.6 ± 0.7, *P* < 0.005); they were more likely to have a higher degree of comorbidity (CIRS score: 6.9 ± 0.9 versus 4.2 ± 0.5, *P* = 0.014) and of disability (lost BADL: 3.7 ± 0.4 versus 0.8 ± 0.2, *P* < 0.001; lost IADL: 5.7 ± 1.0 versus 1.4 ± 0.4, *P* < 0.001), and, as expected, they obtained lower MMSE scores (*P* = 0.001). Patients with unexplained falls were less self-dependent with respect to patients with medical fall causes (lost BADL: 1.4 ± 0.2 versus 2.1 ± 0.4, *P* = 0.016, lost IADL: 2.8 ± 0.4 versus 3.9 ± 0.7, *P* = 0.010) and to patients with dementia-related falls (lost BADL: 1.4 ± 0.2 versus 3.7 ± 0.4, *P* < 0.001; lost IADL: 2.8 ± 0.4 versus 5.7 ± 1.0, *P* = 0.008).

Patients with falls related to medical causes reached higher levels of comorbidity than patients with accidental falls (CIRS score: 7.3 ± 1.0 versus 4.2 ± 0.5, *P* = 0.0007), and they lost a higher number of BADL (2.1 ± 0.4 versus 0.8 ± 0.2, *P* = 0.007) and IADL (3.9 ± 0.7 versus 1.4 ± 0.4, *P* = 0.001). These latter ones referred to a significantly higher number of anamnestic falls in the last year with respect to patients with accidental (*P* = 0.005), dementia-related (*P* = 0.006), and unexplained (*P* = 0.009) falls. Moreover, they showed worse cognitive performances at MMSE with respect to patients with accidental (*P* = 0.006) and unexplained (*P* = 0.030) falls. 

Patients with unexplained falls lost a higher number of IADL with respect to patients with accidental falls (lost IADL: 2.8 ± 0.4 versus 1.4 ± 0.4, *P* = 0.006), and they showed a higher number of depressive symptoms, expressed as GDS score (*P* = 0.020).

No differences were found between the four groups as far as the use of different classes of drugs is concerned.

**Figure 1:**
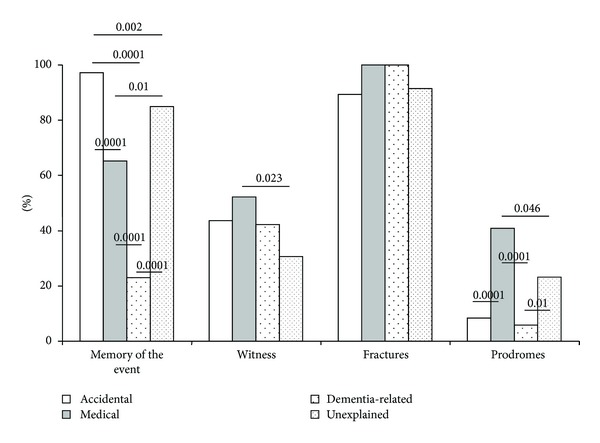
History in different syncope types.

History in different syncope types is illustrated in Figure 1. Patients with accidental falls remember more often the event, as expected. Witness presence is less than 50% in all the fall types. 

## 5. Multivariate Analysis

We drew four multivariate models (logistic regression, method backward stepwise) separately, considering the four fall types as independent variables. We considered in the models the variables that were significantly different between the four groups at the univariate analysis. No predictive factor was found for medical and dementia-related falls. Younger age, low GDS values, and no syncopal spells were independent accidental falls predictors (Table 6(A)), while a higher GDS and syncopal spells were independent predictors of unexplained falls (Table 6(B)). Other variables in the multivariate analysis considered in the model, but not significant, were comorbidity (expressed by means of the Cumulative Illness Rating Score) and the number of lost activities and instrumental activities of daily living. 

## 6. Discussion

According to our knowledge, there is no study about causes of falls leading an old patient to an orthopaedic ward in Italy. Our study demonstrates that these patients are very old and frail because of severe comorbidity and polytherapy. The percentage of patients affected by dementia is quite high (12.6%). The majority of our patients were admitted to hospital because of hip fracture. Hip fractures are very common, and their incidence was not reduced in the last ten years [12]. Moreover 14.8% of patients with hip fractures experienced a second hip fracture in a followup of 4.2 years [13]. For all of these reasons it may be very useful to study the fall etiology to reduce recurrence. 

Our study found a high number of patients with unexplained falls (37%), when the study of Kenny et al. found a significantly lower number of unexplained falls (15%). This difference is explained by the fact that they also considered younger patients (older than 50) admitted to an emergency department, and not to an orthopaedic ward [14]. Unexplained falls can lead to more serious consequences, like hip fractures. Scuffham et al. demonstrated that unspecified falls, although not so frequent as the accidental ones, lead to a significant higher number of hospital accesses and are responsible for 53% of total costs related to falls [15].

A number of different strategies and interventions for each case are effective, but population-based strategies have not yet been evaluated, particularly in frail old patients, admitted to orthopaedic wards. Multidisciplinary, multifactorial intervention programmes inclusive of risk-factor assessment, screening, cause identification by means of diagnostic flow charts, and appropriate intervention proved to be effective [16], and they are useful to identify the causes of fall in the elderly. This topic is mandatory in older patients in order to abolish risk factors and to build a correct prevention programme. Unfortunately we found that only previous syncope and higher GDS score were predictive factors of unexplained falls. For this reason, all patients with fall-related injury must be evaluated for the possible fall cause. A recent meta-analysis showed that in patients with injury-related falls a multifactorial assessment and a targeted intervention do not reduce fall recurrence, whereas the same programme seems to be effective in patients who fall without getting an injury [17]. 

In our “faller” cohort, as shown in Table 3, our patients took a great number of antihypertensive drugs (60.1%) which are well-known fall and syncope risk factors [18]. In a multivariate analysis a previous syncope is a predictor of unexplained falls, while it is a negative predictor of accidental falls. We can speculate that unexplained falls may be caused by syncope more often than normally considered in clinical practice. 

Our study demonstrates the need to study deeply and correctly patients with falls at the very beginning of the story (e.g., when they are admitted to the orthopaedic ward because of the fall). Unfortunately, at the moment, this is very difficult to achieve because of cultural and organizational problems. Future studies may be conducted to evaluate the correct strategy for patients with unexplained falls, probably in a postacute setting such as a rehabilitation unit.

One limitation to this study is the observational design and the absence of an active “prevention and treatment time.” In the literature it is well known that the presence of a team applying comprehensive geriatric assessment and rehabilitation, including prevention, detection, and treatment of fall risk factors, can successfully prevent inpatient falls and injuries, even in those with dementia [19]; this group of old patients is at the highest risk of developing postsurgical complications like delirium [20].

In conclusion, all these data demonstrate that patients admitted to orthopaedic wards after a fall-related injury are frail and affected by severe comorbidity and that unexplained falls are frequent in these patients. These results underline the absolutely relevant role of geriatric evaluation and intervention in older patients admitted to orthopaedic wards. Further studies are necessary to evaluate the impact of diagnostic protocol in patients with unexplained falls. 

## Figures and Tables

**Table 1:** Clinical characteristics.

	All (*n* = 246)	65–79 years (*n* = 79)	≥80 years (*n* = 167)	*P*
Age	82.0 ± 7.0	74.2 ± 4.3	85.7 ± 4.7	0.0001
Sex (males, %)	17.9	21.5	16.2	0.306
Number of drugs	4.2 ± 2.1	4.0 ± 2.2	4.2 ± 2.1	0.569
Use of more than 4 drugs (%)	43.5	43.0	43.7	0.612
CIRS	5.4 ± 4.3	5.1 ± 4.2	5.6 ± 4.4	0.432
Lost BADL	1.6 ± 2.1	0.5 ± 1.3	2.0 ± 2.2	0.0003
Lost IADL	2.5 ± 3.2	1.5 ± 2.5	3.1 ± 3.3	0.001
MMSE	24.6 ± 7.5	27.0 ± 4.4	23.1 ± 8.6	0.003
GDS	4.6 ± 3.3	5.3 ± 3.9	4.0 ± 2.7	0.03
BMI (Kg/m^2^)	24.0 ± 4.1	26.0 ± 5.0	23.3 ± 3.6	0.01
Blood glucose (mg/dL)	112.9 ± 31.1	109.0 ± 27.0	114.5 ± 32.6	0.280
Hemoglobin (g/dL)	11.5 ± 1.7	12.1 ± 1.4	11.2 ± 1.7	0.0004
Creatinine (mg/dL)	1.1 ± 0.9	1.2 ± 1.4	1.0 ± 0.4	0.179

Data are expressed as mean ± standard deviation; CIRS: Cumulative Illness Rating Scale; BADL: basal activities of daily living; IADL: instrumental activities of daily living; MMSE: Mini-Mental State Examination; GDS: Geriatric Depression Scale; BMI: body mass index.

**Table 2:** Clinical history.

	All (*n* = 246)	65–79 years (*n* = 79)	≥80 years (*n* = 167)	*P*
Remember the event	78.9	92.2	72.3	0.002
Witness presence	39.4	45.3	36.6	0.244
Syncope	8.1	7.4	8.3	0.967
Fractures	92.6	90.0	93.9	0.300
Prodromes	17.9	17.7	18.0	0.568

**Table 3:** Drugs taken in the previous month.

	All (*N* = 184)	65–79 years (*N* = 60)	≥80 years (*N* = 124)	*P*
Antihypertensives (%)	60.1	56.7	62.9	0.416
Antiplatelet agents (%)	35.3	26.7	39.5	0.087
Anticoagulants (%)	9.2	15.0	6.4	0.060
Central nervous system drugs (%)	47.5	40.9	50.8	0.208
Ace inhibitors/AT2 antagonists (%)	38.0	38.3	37.9	0.955
Calcium-channel blockers (%)	16.8	18.3	16.1	0.708
Diuretics	34.2	21.6	40.3	0.02
Beta-blockers	13.1	11.7	13.8	0.685
Alpha-blockers	5.4	6.7	4.8	0.608
Other, *n* (%)	79.3	80.0	79.0	0.897

**Table 4:** Different fall types (suggestive diagnosis).

	All (*n* = 246)	65–79 years (*n* = 79)	≥80 years (*n* = 167)	*P*
Accidental (%)	99 (40.2)	38 (48.1)	61 (36.5)	0.02
Medical (%)	25 (10.2)	7 (8.9)	18 (10.8)	0.323
Dementia-related (%)	31 (12.6)	5 (6.3)	26 (15.6)	0.02
Unexplained (%)	91 (37.0)	29 (36.7)	62 (37.1)	0.475

Data are expressed as number (percentage).

**Table 5:** Clinical patient features with different fall types.

	Accidental (*N* = 99)	Medical (*N* = 25)	Dementia-related (*N* = 31)	Unexplained (*N* = 91)
Age (years)	80.6 ± 0.7	82.2 ± 1.4	85.9 ± 1.2	82.4 ± 0.7
Sex (males, %)	14.1	24.0	9.7	23.1
Number of falls	1.7 ± 0.3	3.5 ± 0.5	1.6 ± 0.4	1.9 ± 0.3
Number of drugs	3.8 ± 0.2	4.1 ± 0.5	4.6 ± 0.4	4.3 ± 0.2
More than 4 drugs (%)	38.3%	44.0%	51.0%	46.1%
CIRS	4.2 ± 0.5	7.3 ± 1.0	6.9 ± 0.9	5.5 ± 0.6
Lost BADL	0.8 ± 0.2	2.1 ± 0.4	3.7 ± 0.4	1.4 ± 0.2
Lost IADL	1.4 ± 0.4	3.9 ± 0.7	5.7 ± 1.0	2.8 ± 0.4
MMSE	26.1 ± 0.9	20.6 ± 1.8	14.0 ± 3.6	25.0 ± 0.1
GDS	3.8 ± 0.4	4.5 ± 0.9	5.0 ± 2.3	5.3 ± 0.4
BMI (Kg/m^2^)	24.2 ± 0.6	26.0 ± 1.3	20.4 ± 1.8	24.0 ± 0.8
Blood glucose (mg/dL)	109.4 ± 3.7	121.3 ± 6.6	108.8 ± 6.5	115.0 ± 3.7
Hemoglobin (g/dL)	11.7 ± 0.2	11.8 ± 0.4	11.1 ± 0.3	11.3 ± 0.2
Creatinine (mg/dL)	0.9 ± 0.1	1.4 ± 0.2	0.9 ± 0.2	1.2 ± 0.1

Data are expressed as mean ± standard error or %; CIRS: Cumulative Illness Rating Scale; BADL: basal activities of daily living; IADL: instrumental activities of daily living; MMSE: Mini-Mental State Examination; GDS: Geriatric Depression Scale; BMI: body mass index.

**Table 6:** Multivariate analysis: types of fall predictors.

	OR	95.0% CI	*P*
(A) Independent factor: accidental fall			
Age	0.66	0.45–0.98	0.05
GDS	0.63	0.45–0.89	0.01
Syncopal spells (anamnestic)	0.59	0.43–0.83	0.005

(B) Independent factor: unexplained fall			
GDS	1.49	1.06–2.09	0.029
Syncopal spells (anamnestic)	1.49	1.04–2.12	0.036

GDS: Geriatric Depression Scale.

## Appendix

### Centers and Investigators Participating to the Study

1. Florence, Syncope Unit, Department of Geriatric Cardiology, University of Florence and Azienda Ospedaliero Universitaria Careggi. Investigators: Andrea Ungar, Annalisa Landi, Alice Maraviglia, Niccolò Marchionni, Giulio Masotti, Alessandro Morrione, and Martina Rafanelli.
2. Modena, Chair of Geriatrics, University of Modena and Reggio Emilia: Chiara Mussi, and Gianfranco Salvioli.
3. Trento, Division of Geriatrics, Santa Chiara Hospital: Gabriele Noro, and Gianni Tava.
4. Reggio Emilia, Division of Geriatrics, Santa Maria Nuova Hospital: Loredana Ghirelli.
5. Naples, Department of Geriatrics, Federico II University: Pasquale Abete, Vincenzo Del Villano,  Gianluigi Galizia, and Franco Rengo.
6. Grosseto, Division of Geriatrics, Walter De Alfieri, Fabio Riello.
7. Chiavari, Department of Geriatrics, Paolo Cavagnaro.
